# Parkinson's Disease and the Environment

**DOI:** 10.3389/fneur.2019.00218

**Published:** 2019-03-19

**Authors:** Nicole Ball, Wei-Peng Teo, Shaneel Chandra, James Chapman

**Affiliations:** ^1^School of Health, Medical and Applied Sciences, Central Queensland University, Rockhampton, QLD, Australia; ^2^School of Exercise and Nutrition Sciences, Institute for Physical Activity and Nutrition, Deakin University, Melbourne, VIC, Australia; ^3^Physical Education and Sports Science Academic Group, National Institute of Education, Nanyang Technological University, Singapore, Singapore; ^4^School of Science, RMIT University, Melbourne, VIC, Australia

**Keywords:** Parkinson's disease, demographical, environmental, pesticides, heavy metals, illicit drugs

## Abstract

Parkinson's disease (PD) is a heterogeneous neurodegenerative disorder that affects an estimated 10 million sufferers worldwide. The two forms of PD include familial and sporadic, and while the etiology of PD is still largely unknown, the condition is likely to be multifactorial with genetic and environmental factors contributing to disease genesis. Diagnosis of the condition is attained through the observation of cardinal clinical manifestations including resting tremor, muscle rigidity, slowness or loss of movement, and postural instability. Unfortunately, by the time these features become apparent extensive neurological damage has already occurred. A cure for PD has not been identified and the current therapy options are pharmaceutical- and/or surgical-based interventions to treat condition symptoms. There is no specific test for PD and most diagnoses are confirmed by a combination of clinical symptoms and positive responses to dopaminergic drug therapies. The prevalence and incidence of PD vary worldwide influenced by several factors such as age, gender, ethnicity, genetic susceptibilities, and environmental exposures. Here, we will present environmental factors implicated in sporadic PD onset. By understanding the mechanisms in which environmental factors interact with, and affect the brain we can stride toward finding the underlying cause(s) of PD.

## Introduction

Parkinson's disease (PD) is characterized as a progressive neurodegenerative disorder that results in death (1) and affects ~1–3% of the global population aged over 60 years (2, 3). There are two forms of PD, familial; genetically inherited in either an autosomal dominant or recessive manner (4), and sporadic (idiopathic); believed to develop from gene-environment interactions (5). Genetically linked PD accounts for ~10–15% of all PD cases (6) with the remainder classed as sporadic. Seven causal genes have been identified for familial PD; alpha-synuclein (SNCA), glucocerebrosidase (GBA), leucine-rich repeat Kinase 2 (LRRK2), vacuolar protein sorting-associated protein 35 (VPS35) parkin RBR E3 ubiquitin protein ligase (PARK2), phosphatase and tensing homolog-induced Kinase 1 (PINK1), and Parkinson protein 7 (PARK7) (6, 7). These genes, along with specific metabolites (8) and PD-associated biomarkers (9–11) have been used to research possible early detection methods for PD. Due to an estimated 70% of neuronal death before the onset of clinical symptoms (12) early diagnosis of PD is essential.

Gene-environment interactions are understood to be the underlying cause of idiopathic PD. Human genotypes are independently unique and individuals exposed to the same environmental factor are affected differently, resulting in diverse disease phenotypes (13). The combined effect of genetic and environmental factors may influence the onset of human disease (14) by structurally altering deoxyribonucleic acid (DNA) (15). For example, caffeine is an adenosine A2A (ADORA2A gene) receptor antagonist which increases dopamine neurotransmission (16) and polymorphisms of ADORA2A have been found to reduce the risk of PD (16, 17). In contrast, pesticides and heavy metals display a negative effect and increase PD by causing gene variations linked to familial PD (e.g., PARK1, LRRK2, PINK1) resulting in PD-associated mechanisms such as mitochondrial dysfunction, oxidative stress and protein degradation impairment (18, 19).

Demographic factors including age, gender and ethnicity may also impact PD susceptibility. Age is a major risk factor for PD (20). Not only has the prevalence of PD been observed with increasing age (21), but so too the severity of disease symptoms (22). Most cases develop between the ages of 60–65 years however, young on-set (<50 years) and juvenile cases (<21 years) have also been identified (23). Gender may also predispose an individual to PD. The incidence of PD amongst men is higher than women (14). Estrogen may act as a neuro-protective agent (24), and women who have had a high lifetime exposure from such things as lengthy fertility windows and multiple births show a reduced risk of developing PD (25, 26).

The prevalence of PD due to ethnicity is sparse. Recent research has highlighted predominantly white Western populations in having higher PD prevalence than Asian nations (27). Older studies have shown a correlation between Caucasians and Hispanic races having an increased risk of developing PD when compared to Asian and Black cultures (28, 29). One reason for this could be, commonly, individuals with darker skin tones produce more melanin than fair skinned people (30), which may suggest increased levels of neuromelanin in the substantia nigra may have a neuro-protective effect. Another explanation lies in the socioeconomic state of a nation. Industrialized countries have higher urbanization leading to environmental degradation and exposure to environmental toxins (31). Research has shown areas in which socioeconomic status is higher equates to an increased incidence of PD (32).

Due to the diverse nature of the disease, its symptomology differs from one individual to the next. Some cases may be influenced by demographical factors such as age, gender, and ethnicity while others may be attributed to environmental, occupational, or residential exposure to neurotoxins which may selectively target substantia nigra neurons (33) including, heavy metals, pesticides and illicit drugs. This mini-review will focus on environmental factors which have been implicated in PD.

## Environmental Contributors

Numerous environmental toxins have been implicated in the onset of PD, however, data has been inconsistent. Some studies suggest PD incidence may be linked to occupational exposure to chemicals (34–36). Several occupations, some with increased risk have been investigated, for example, agriculture, and working with pesticides (37) and heavy metals (38). Others having a null effect, such as electrical vocations, and working with extremely low frequency-magnetic fields (39), diesel motor emissions, or solvents (40) have also been examined. Rural residency as a causative factor for idiopathic PD has been a long-disputed topic amongst PD research. Some studies have found no link between rural living and PD, citing the opposite that urban living may lead to an increased risk (28, 41). Studies conducted on highly populated urban areas have also found significant correlations between industrial airborne heavy metal pollution (42, 43) and ambient air pollution from traffic (44, 45), and an augmented chance of PD onset. Other studies have found no difference between geographical location and the inflation of PD incidence (46, 47). Still, other investigations have associated rural exposure as a heightened risk (34, 48, 49). Elevated incidences of PD in rural locations may be attributed to these locations having a proportionately higher number of aged population (50).

### Heavy Metals

Neurotoxins in the brain can lead to oxidative stress and neurotransmission disruption with detrimental effects in the basal ganglia (51). The generation of reactive oxygen species occurs when hydroxyl radicals are produced from hydrogen peroxide under the Fenton-Haber-Weiss reaction (52). This reaction may lead to oxidative stress (53) and neurotoxicity (54) thus, causing damage to numerous aspects of the cell with preferential damage to the mitochondria (55). Hydroxyl radicals react to deoxyribonucleic acid (DNA), membrane lipids, and proteins of the cell leading to their eventual dysfunction (51). Within the neuron, the reduction and metabolism of dopamine results in the generation of hydroxyl radicals as by-products, thus resulting in a ready supply of hydroxyl radicals within the substantia nigra pars compacta (51).

**Figure 1:**
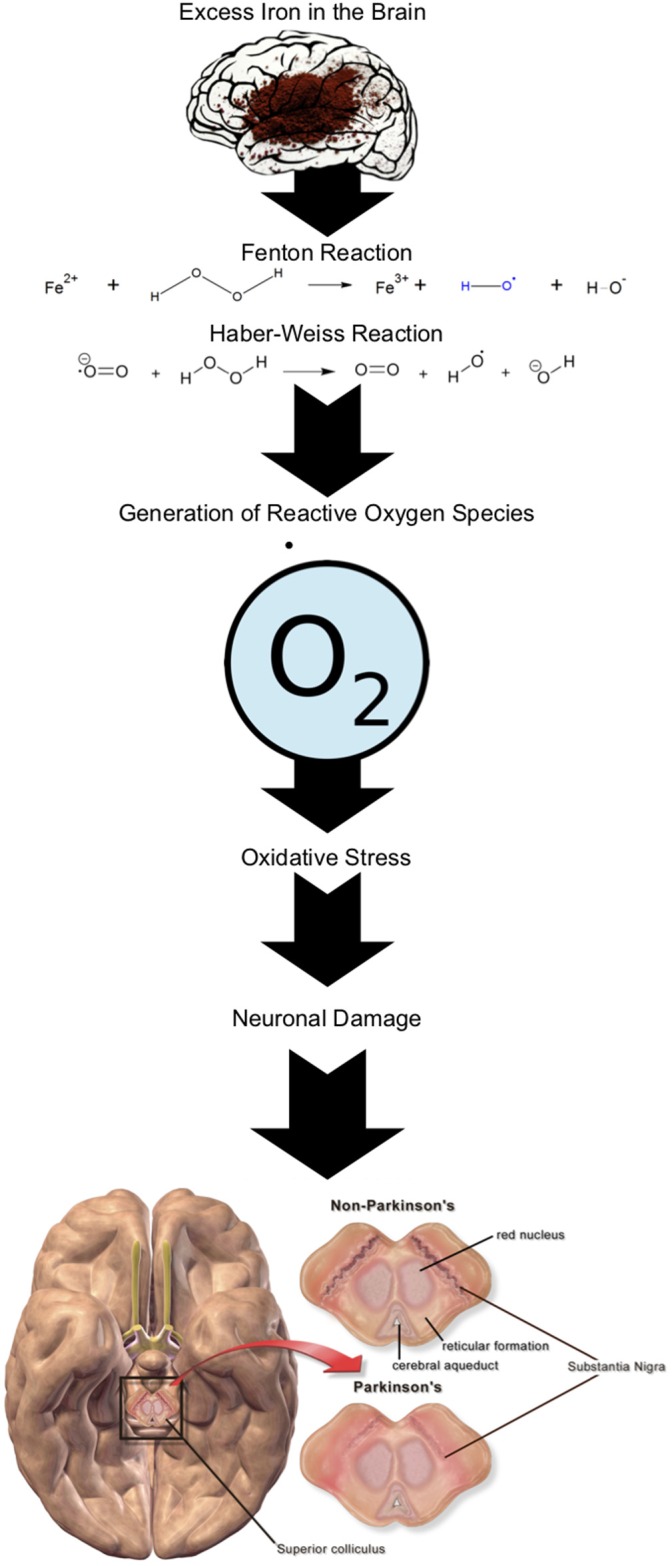
Fenton-Haber-Weiss reaction in the presence of Fe in the brain generating reactive oxygen species leading to neuronal damage (${\text{O}}_{2}^{•-}=$ superoxide anion radical).

Numerous studies deny any relationship between PD etiology and heavy metals (56–60), while others have claimed an association (61–63). Iron is one heavy metal implicated due to the role it plays in the generation of oxidative stress (via the Fenton-Haber-Weiss reaction, Figure 1) leading to neuronal death (52), as well as its involvement in alpha-synuclein toxicity (51). A meta-analysis of five studies including 126, 507 PD individuals found no significant link between dietary iron and an increased risk of PD [moderate iron intake; relative risk [RR] 1.08, 95% CI 0.16–1.93, *p* = 0.787 and high iron intake; RR 1.03. 95% CI 0.83 – 1.80, *p* = 0.766] (56). This research indicates dietary intake of iron does not proliferate PD risk (56) giving more credibility to environmental pathways as vectors of susceptibility. The meta-analysis was robust in its design with stringent selection criteria. A limitation was differing methodology between the studies. Four studies used recent recall techniques whereas, the fifth used a past-history approach which may have resulted in recall bias.

Similar to iron, copper contributes to oxidative stress however copper has two modes of action; the Fenton-Haber-Weiss reaction (55) and 6-OHDA (6-hydroxydopamine) redox cycle (62). Accumulation of copper in the brain has seen the reduction of dopamine (64), alpha-synuclein aggregation (61) and the reduction of the protective factor for neuronal survival; superoxide dismutase 1 (64). Research shows that occupations which expose an individual to copper intensifies a PD risk (65). Gorell et al. conducted a 20-year investigation into the link between occupational heavy metal exposure and PD and found an individual with chronic copper exposure had a two-and-a-half-fold increased risk of PD [Odds Ratio [OR] 2.49, 95% CI 1.06–5.89] (65). To measure occupational exposure this study used an extensive “risk factor questionnaire.” Self-reporting questionnaires run the risk of recall bias. To minimize this, the investigators employed an experienced industrial hygienist blinded to a participant's status to assess job description concentrations. One flaw in the study design is, the researchers did not measure heavy metals concentrations within participating individuals which could have further supported their claims.

Manganese may amplify PD risk through manganese toxicity resulting in impaired motor function and damage to the substantia nigra and other basal ganglia nuclei (66). Exposure can occur through diet, occupation and environmental pathways (51, 67). Chronic manganese intoxication can clinically resemble idiopathic PD (68). Manganese-induced Parkinsonism may be identified due to its predilection to accumulate in and damage the palladium and striatum as opposed to the substantia nigra pars compacta in PD (69).

Lead exposure may see a two-to-three-fold increased risk of PD. Coon et al. (70) and Weisskopf et al. (71) used K-X-ray fluorescence on tibia bone (PD patients vs. controls) and both found an increased risk of PD in individuals with chronic lifetime exposure to lead; OR 2.27, 95% CI 1.13–4.55, *p* = 0.021 and OR 3.21, 95% CI 1.11–5.93, *p* = 0.03, respectively (70, 71). Bone measurement of Pb is a reliable tool as it is able to account for more than 90% of cumulative Pb burden in adults (72) when compared to blood measurement which can only give a result for recent exposure (4–6 weeks) (73). Additionally, the Coon et al. study strengthened their data by combining occupational exposure (assessed by an industrial hygienist) and blood Pb levels with their Pb bone results. Lead is able to enter the brain by mimicking calcium via calcium channels (70). Monnet-Tschudi et al. (74) exposure to lead can cause several neurological deficits including severe swelling and loss of neurons in the central nervous system (CNS) and peripheral nervous system, manifesting as motor-based dysfunction in the form of loss of voluntary muscle movement (51). Occupational exposure to lead, especially in the long-term, may increase concentration levels of lead within subjects, heightening PD risk (70, 71).

Mercury is a known neurotoxin which can cause neuronal death (75). Research is still conflicted regarding its participation in PD pathogenesis, however, mercury is present in organic (elemental mercury and methylmercury), and inorganic forms (51, 76) and can contaminate water sources including rain, ground, and sea. In the aquatic environment, inorganic mercury is the most toxic form when it is microbially transformed into methylmercury. The transformation also makes mercury prone to bioaccumulation and biomagnification leading to a potential food web transfer causing mercury-laced meat, vegetables, and fish (77). It has been reported mercury can reduce the number of neurons present in the brain (75) and cause movement disorders including tremors and loss of voluntary muscle movement (51).

### Pesticides

In the 1980's interest in the action of 1-methyl-4-phenyl-1,2,3,6-tetrahydropyridine (MPTP) arose due to its ability to selectively target the substantia nigra. Patients who had been exposed to MPTP presented with textbook cases of advanced PD. The research discovered MPTP was metabolized by astrocytes in the brain to 1-methly-4-phenylpyridium (MPP+) which had a distinctively similar composition to a popular pesticide; paraquat thus, inciting the hypothesis of pesticides and their role in PD pathogenesis (78). The current evidence suggests there is an association between some pesticides and PD (Table 1).

**Table 1:** Risk factors of PD in the use of pesticides (with confidence interval of 95%).

**Reference**	**Pesticide(s)**	**Cases vs. controls**	Odds ratio^*^
Tanner et al. (79)		110 vs. 358	
	Paraquat	23:49	2.50 (1.40–4.70)
	Rotenone	19:32	2.50 (1.30–4.70)
Wang et al. (80)		362 vs. 341	
	Paraquat	81:78	1.26 (0.86–1.86)
	Ziram	6:6	1.37 (0.42–4.49)
	Maneb and Paraquat	26:21	1.41 (0.75–2.68)
	Ziram and Paraquat	37:24	1.82 (1.03–3.21)
	Ziram, Maneb, and Paraquat	46:18	3.09 (1.69–5.64)
Dick et al. (81)		959 vs. 1989	
	Any exposure to pesticides		1.25 (0.97–1.61)
	Low vs. no exposure		1.09 (0.77–1.55)
	High vs. no exposure		1.39 (1.02–1.89)

* *Odds Ratio (OR): a ratio for the measure of association between exposure and outcome; OR = 1 exposure has no effect on outcome, OR > 1 exposure associated with higher risk of outcome, OR < 1 exposure associated with lower risk of outcome (82)*.

Pesticides (including insecticides and herbicides) are used globally in public health to control disease vectors and in the agricultural industry to control pests and their subsequent diseases (83, 84). They have been consistently investigated due to their ability to affect neurological changes in the brain leading to the destruction of dopamine producing neurons (85). Insecticide classes commonly investigated include organochlorines and organophosphates. A well-known organochlorine pesticide associated with increasing the risk of PD is dieldrin which affects the CNS causing neurotoxic damage to the dopaminergic system (86). Two recent studies on mice models have confirmed preferential targeting of the dopaminergic system by dieldrin (87, 88) and both found the severity of the impact of dieldrin was dose-dependent. Rotenone is the major organophosphate constituent for raising PD risk and its mode of action is the acceleration of alpha-synuclein aggregation resulting in the death of dopaminergic neurons (89). Like dieldrin, its effects appear to be dose-dependent (90, 91). The question remains, are non-human models able to predict human health effects with enough efficacy? Correlation between animal models and human disease is only an estimated 60% (92).

PD associated risk and herbicide exposure are still unclear. Studies have found some correlation between PD risk and the use of a combination of herbicides (93) and occupational exposure (80) however, individual herbicide action has not been identified as causative. Tanner et al. conducted an occupational study and reported pesticide use, rather than occupation was a risk factor for PD (93). 2,4-Dichlorophenoxyacetic acid (2,4-D) a primary constituent in Agent Orange was identified as being statistically significant risk for PD (OR 2.59, 95% CI 1.03–6.48, *p* = 0.04). Also noted was the significant association between PD and exposure to any 1 of 8 specific pesticides (2,4-D, paraquat, permethrin, dieldrin, diquat, maneb, mancozeb, and rotenone) OR 2.20, 95% CI 1.02–4.75, *p* = 0.04. Hancock et al. also found a positive correlation for 2,4-D however, the association was not statistically significant (94). Other studies concluded no association between herbicides and increased PD risk (95, 96).

Many pesticide studies have looked at human exposures through population-based studies which can suffer from bias. These biases may occur in the forms of (a) recall bias with over- or under-estimation of exposure amount or time, (b) social bias where public approval may influence responses on sensitive topics (e.g., illicit drug use underestimation), (c) measurement error bias including systematic and random error, and (d) confirmation bias where a patient's preconceptions or beliefs can influence their response (97).

### Illicit Substances

A history of illicit drug use has been found to cause abnormal morphology in the substantia nigra (98), such that, substantia nigra hyperechogenicity is a major risk factor for the development of PD (99). The abuse of illegal stimulants can elevate reactive oxygen species levels causing oxidative stress leading to dopamine neuron toxicity and death (100). Illicit drug residues have been found in river basins, surface waters, and wastewaters, with inadequately-treated municipal wastewater discharge, cited as the main vector of contamination (101). Water epidemiology studies have been used in recent times to estimate community drug use by measuring drug residues in waste-water (102–104). These studies have succeeded in the detection and estimation of drug residue concentrations in wastewater. Analysis of drinking and surface waters has found extrication rates of illicit drugs are inadequate, with results demonstrating detectable levels after treatment (101, 105–107). Due to the high production volume and use of illicit drugs, their residues may persist in the environment, and leach into other environmental media including sediments and biota (107, 108).

The three common psychostimulants implicated in PD are amphetamine, methamphetamine and cocaine (109–111). High doses of the stimulant drug amphetamine may cause damage to dopaminergic neurons and axon terminals within the human brain (112). Animal studies on non-human primates and rodents have found acute amphetamine exposure is toxic to dopamine terminals (113). Garwood et al. (109) through a telephone survey found the prolonged use of amphetamine may be a statistically significant risk factor for PD (OR 4.46, 95%CI 1.0–19.8, *p* = 0.049). This study had some limitations such as possible recall bias due to the human factor of the survey, a small sample size (*n* = 76 PD), and it did not distinguish between prescribed and non-prescribed amphetamine use. This is an important differentiation to be assessed as amphetamines are also used to treat PD (109).

In addition, methamphetamine, a highly water-soluble psychostimulant drug plays a role in decreasing the integrity of dopamine neuron terminals in the basal ganglia, reducing levels of dopamine, and dopamine transporters (114, 115). These effects can be long-term, causing damage to the dopamine terminal and death of dopamine neurons (114, 116, 117). A cohort study using hospital admission records found methamphetamine/amphetamine exposure increased PD risk nearly three-fold when compared to unexposed controls (Hazard Ratio 2.8, 95%CI 1.6–4.8, *p* = 0.01) (111). Although the study had a good sample size (PD *n* = 4935, control *n* = 24,675), due to no “gold standard” of PD diagnosis some members of the control group may have been undiagnosed. Additionally, researchers did not account for other medications or addictions (e.g., alcohol) which may cause PD-like symptoms.

Lastly, cocaine is a drug of abuse known to bind to dopamine transport proteins causing short-term inhibition of dopamine uptake (118, 119) however, no direct association has been found between an elevated risk of PD and cocaine use (110, 111). A study of 44 cocaine users vs. 44 controls medical records reported cocaine users showing excess iron accumulation in the Globus pallidus. This suggested cocaine addiction may lead to iron dysregulation (120) thus, possibly contributing further to oxidative stress. Although the effects of illicit drugs and their role in PD pathophysiology presently remain unclear, results so far have shown a strong argument for a possible role in neurodegeneration. Further continued research is warranted to fully understand these roles and associations.

## Conclusion

In the 200 years since James Parkinson first described the “shaking palsy” phenomenon, significant research in roads toward diagnosis and causative associations with PD have been realized. Yet, science has still not been able to deduce the complete etiology of PD. There have, however, been many advances in the genetic inheritance of the disease. The role in which environmental factors interact to contribute to the pathophysiology of the disease remains elusive, however, as discussed, many demographical/environmental factors may be at play in the etiology of PD and may impact the severity of the disease. Age is a major risk factor for the condition, however, age alone may not be the only contributor to PD genesis. Gender, ethnicity, living circumstances, and occupation have been implicated in heightened PD risk nevertheless, data on this is still contradictory. Study design can also impact the reliability of data and reporting of causal links to PD. Using a mixed-method approach encompassing patient history as well as chemical analysis could improve study robustness especially in regards to population-based studies. Exposure to toxins in the environment has been linked to PD-associated neurodegeneration particularly heavy metals, pesticides, and illicit drugs. These avenues of exposure can be directly related to rural living, adding support to the argument that rural living may be a risk factor for PD itself. Each case of PD is specific to the individual and given the heterogeneity of the disease, one can hypothesize that individual susceptibility to environmental factors plays a large role in PD etiology. The complex nature of PD only adds to the difficulty in pinpointing its cause.

## Author Contributions

Manuscript conceived by NB, W-PT, SC, and JC. Initial drafting completed by NB. Contributed and critically reviewed by NB, W-PT, SC, and JC. All authors read and approved the final manuscript.

### Conflict of Interest Statement

The authors declare that the research was conducted in the absence of any commercial or financial relationships that could be construed as a potential conflict of interest.
